# Application of CT images based on the optimal atlas segmentation algorithm in the clinical diagnosis of Mycoplasma Pneumoniae Pneumonia in Children

**DOI:** 10.12669/pjms.37.6-WIT.4860

**Published:** 2021

**Authors:** Xilin Fu, Ningfei Yang, Jianwei Ji

**Affiliations:** 1 Xilin Fu, Attending Physician, Department of Pediatrics, Yiwu Central Hospital, Yiwu, 322000, China; 2 Ningfei Yang, Attending Physician, Department of Pediatrics, Yiwu Central Hospital, Yiwu, 322000, China; 3 Jianwei Ji, Attending Physician, Department of Pediatrics, Yiwu Central Hospital, Yiwu, 322000, China

**Keywords:** Clinical Diagnosis, CT Images, Children, Optimal Atlas Segmentation Algorithm, Mycoplasma Pneumoniae Pneumonia

## Abstract

**Objective:**

Use of optimal Atlas segmentation algorithm to study the imaging signs of mycoplasma pneumonia with multi-slice spiral CT (HRCT), and to explore the value of HRCT in the diagnosis and efficacy in evaluation of mycoplasma pneumonia in children.

**Methods:**

The study retrospectively analyzed 72 patients diagnosed with mycoplasma pneumonia in our hospital from January 2017 to January 2019. The imaging data and clinical data of 72 patients were collected. The optimal Atlas segmentation algorithm was used to analyze the characteristics of CT examination, and the value of CT in the diagnosis of mycoplasma pneumonia and the evaluation of curative effect was summarized.

**Results:**

Among all patients, 37 cases were unilateral lesions, 35 cases were bilateral lesions, 19 cases were in the left upper lobe, 24 cases were in the left lower lobe, 21 cases were in the right upper lobe, 13 cases were in the right middle lobe, 25 The lesion was located in the right lower lobe. The main CT findings of the lesions before treatment were large patchy, spot-shaped shadows, and strip-shaped or ground-glass shadows. After treatment, the main CT findings of the lesions were reduced lesion density and reduced lesion range.

**Conclusion:**

CT can clearly show the pulmonary lesions of mycoplasma pneumonia, and its unique imaging signs can improve the clinical diagnosis accuracy. In addition, CT scans can evaluate the treatment effect according to the changes in the characteristics of the lesion, which has important value for the evaluation of the effect for clinical diagnosis and efficacy evaluation of mycoplasma pneumonia.

## INTRODUCTION

Mycoplasma pneumoniae cause serious complications in other systems of the body, such as varying degrees of damage to the cardiovascular, digestive system, skin, immune system, and nervous system.^1^,^2^ Related studies have also shown that mycoplasma has a degree of correlation with cough variant asthma and the acute attack of asthma.^3^ Children infected with mycoplasma probably have chronic cough and cough variant asthma.^4^,^5^ The infection of Mycoplasma is not only limited to the infection of the respiratory tract, as it has invaded other multi-system infections, causing serious complications such as septic shock.^6^ The proportion of mycoplasma infection has further increased. To avoid further aggravation of infection, early treatment of mycoplasma infection in children is particularly important.^7^

Patients with mycoplasma pneumonia have severe symptoms. Mycoplasma pneumonia has a long course, but patient can completely recover after long-term treatment.^8^ Our objective was to study the unique imaging signs of spiral CT in mycoplasma pneumonia, its value in diagnosis and evaluation of curative effect .

## METHODS

Seventy-two patients diagnosed with mycoplasma pneumonia through pathological and pathogenic examinations were included in this study performed from January 2017 to January 2019 are studied in our hospital after IRB approval (dated March 21, 2021).

###  Inclusion criteria

(1) The patient was diagnosed with mycoplasma pneumonia after pathogenic examination and imaging examination; (2) All the selected children had complete imaging, pathogenic examination, and pathology; (3) The selected patient was 70 years old > Age > 4 months.

### Exclusion criteria

(1) Persons with missing data; (2) Patients and their family members unwilling to sign the relevant informed consent; (3) thirty-four were male patients and 38 were female patients. 65% were under 13 years of age, with an average age of (23.13±2.04) years old, a course of 4~16 days, and an average course of (7.83±). 3.25) days was administered. The clinical manifestations of all patients were fever, anorexia, cough, chills, headache, sore throat, substernal pain and other symptoms, with fever and cough as the main manifestations.

### Equipment and inspection methods

All 72 patients were scanned by multi-slice spiral CT (MSCT). The Optima CT 520 Kunlun 16-slice spiral CT machine in our hospital was used for scanning. The lung was completely scanned. Scanning parameters: tube voltage 120KV, tube current 160mA, scanning pitch 0.875:1, layer thickness 5mm, layer distance 5mm, matrix 512×512, both mediastinal window (window width 450Hu, window level 45Hu) and lung window (window the width is 1000 Hu, and the window is 2500 Hu). Thin-layer scanning was performed, and adults had an enhanced scan. Iohexol contrast agent was injected into the patient’s vein at a speed of 1.5-3.0 mL/s, and the scan was started 18-30 seconds after the injection of iohexol.

### Optimal Atlas image selection strategy

Transform the tissue images between different individuals at a specific scale, and conduct depth analysis and appropriate orientation of their internal structures. The shapes and sizes of the tissue anatomical structures have commonality. Empirical knowledge performs accurate registration-segmentation of the image to extract the image ROI.

Construct a database by labeling a large number of medical template images *p*(1,2,...,*n*). n is the number of template images in the database *Atlas*. Fixed atlas subsets and matching images are used to select the best atlas set for multiple atlas segments. Randomly sampled image subsets are proposed, combined with perceptual hash matching to select the best *Atlas* image, and a multi-atlas hierarchical registration method with higher registration accuracy is adopted. First mark the position of each atlas image p as *p*_*LandMark*(1,2...,*d*)´_, and the position of each layer image q of the input 3D lung medical image to be registered as *q*_*LandMark*(1,2...,*d*)´_, the image to be registered is based on the atlas image layered with the minimum *p*_*LandMark*(1,2...,*d*)_ value. Quasi, *p*_*LandMark*(1,2...,*d*)_ is expressed as:


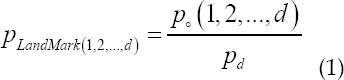


_o_ represents the current image in the three-dimensional image; d is the total number of images in the three-dimensional image. Label the input 3D lung image data with label *p*_*LandMark*_, find the nearest *p*_*LandMark*_ atlas as the optimal *Atlas* image for registration, and obtain the mapping space transformation parameters. For the corresponding mark image *L*(1,2...,*d*) in the atlas, the mapping space transformation generated based on the registration is generated the mark map *L′*(1,2...,*d*) of the image to be registered, finally, image fusion is performed on all the layered mark images to obtain the image segmentation result.

Registration-segmentation depends on the similarity of two images. Choosing a registration framework needs to estimate the image mapping relationship. To reduce the amount of calculation, the image to be registered and the atlas set are aligned. This process is the search process of the optimal mark image, and it is based on the Land Mark image matching method of randomly sampled image subsets combined with perceptual hashing to realize the fast search of the optimal atlas set of three-dimensional registered images based on optimal similarity matching.^9^ The optimal similarity *S(t)* is determined by *p_LandMark_*+Δ_*t*_ and the hash function H:


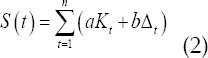


Δ_*t*_ is the deviation value of *p*_*LandMark*_; *K_t_* is the hash similarity; and t is the current registration template image. Through experiments, *a* = 0.25,*b* = 0.75 it is concluded that in the mark atlas *L*(1,2,...*n*), when Δ_*t*_ is 0 to 0.02, and is greater than 0.85, the effect of image registration is better. According to the *S(t)* value, the atlas images are optimally sorted according to matching similarity.

B-spline free transformation is a key non-rigid deformation registration transformation type in image processing. This scheme improves the method, and uses its registration transformation model as a local weighted two-dimensional gridding collator [10]. The B-spline non-rigid transformation model is a combined transformation model composed of global component *T_global_(x)* and local component *T_local_(x)*:


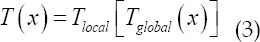


x is the image transformation unit. In this scheme, a regular two-dimensional grid Φ model with uniformly spaced *i,j*(0≤*i*≤*c_x_*,0≤*j*≤*c_y_*) and *c_x_* × *c_y_* grid control points are set in the x and y directions. After the image to be registered and the reference image are gridded, moving the grid control points causes the neighborhood grid the pixel points of will also change^11^, so that the image will be deformed accordingly, and the amount of image change information can be expressed as:


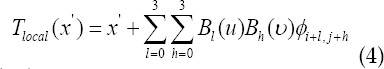



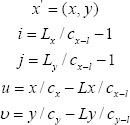
 Represents the lth third-order basis function of the B-spline in the x direction; 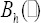 represents the hth third-order basis function of the B-spline in the y direction; L is the distance of the grid movement in the current direction. The above u and 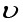 are the derivative variables of the deformation function in the x and y directions respectively, the same expression can be used, and the coefficient of the deformation function can be obtained by derivation:


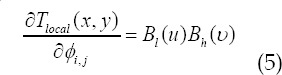


In the formula: *l = i-Lx/n_x-1_+1;h=j-Ly/c_y-1_+1*. When 0 < l < 2 , *B_l_(u)* = 0. The derivative is non-zero in the neighborhood of a given point, and the objective function is optimized based on the gradient descent method.

## RESULTS

After integrating the CT imaging diagnosis results of 72 patients showed that 37 were unilateral lobe lesions and 35 bilateral lobe lesions; 19 cases were located in the upper left lobe, 24 in the left lower lobe, and 21 cases in the upper right lobe, 13 lesions were in the right middle lobe, and 25 lesions in the right lower lobe. The main CT findings of the lesion are large patchy and speckled shadows, and the lung texture of the child is increased, and the shadow is striped or ground glass; 56 of the 72 patients showed thickening of the bronchial wall eight cases showed enlarged hilar and mediastinal lymph nodes, two cases showed signs of cavitation, and nine cases had pleural effusion. Table-I.

**Table-I:** CT diagnosis results of 72 patients [n (%)].

		*No. of cases*
Lesion distribution	Unilateral lung disease	37(51.39)
	Right middle lobe	13(18.06)
	Right upper lobe	21(29.17)
	Right lower lobe	25(34.72)
	Upper left lobe	19(26.39)
	Left lower lobe	24(33.33)
	Bilateral lobe lesions	35(48.61)
CT image features	Large patchy shadows	63(87.50)
	Spotty shadow	28(38.89)
	Increased lung texture	24(33.33)
	Cord shadow	15(20.83)
	Ground glass shadow	11(15.28)
Other lung manifestations	Bronchial tube wall thickening	56(77.78)
	Swollen hilar and mediastinal lymph nodes	8(11.11)
	Hollow sign	2(2.80)
	Pleural effusion	9(12.50)

A 6-year-old female child came to the clinic with a cough and fever. Laboratory test results showed positive for mycoplasma culture and positive for mycoplasma antibodies. After CT examination, the diagnosis was mycoplasma pneumonia. The CT signs before treatment were: multiple patchy lesions in the anterior and posterior apical segments of the left upper lung lobe, mainly manifested as multiple small patchy high-density shadows with uneven density and blurred edges, visible tree buds, and bronchial wall thickening. Lung window display showed small patches of increased density which could be seen in the anterior segment of the left upper lung, in the form of tree buds, the bronchial tube wall is thickened, and the lung texture was thickened and thickened, showing a cord-like shadow.

**Fig.1:**
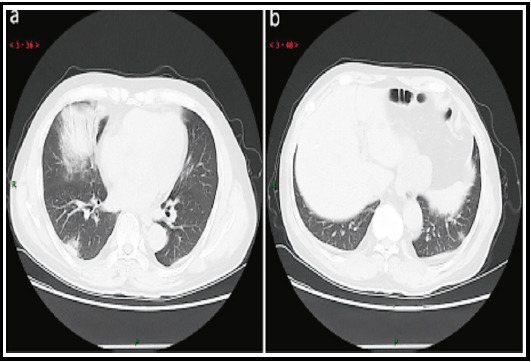
CT image of mycoplasma pneumonia after treatment. The absorption of patchy shadow in the upper left lobe of the lesion is reduced after treatment

Seven days later, the CT findings showed that the lung window showed multiple small patchy high-density shadows in the anterior and posterior segments of the left upper lung lobe, and the area of the lesion was reduced. After the treatment of the left upper lung lobe, the patchy shadow absorption decreased, as shown in Fig.1. The mediastinal window is basically normal.

## DISCUSSION

Seventy-two patients with Mycoplasma pneumoniae were selected as the research objects in this study, and all patients underwent HRCT examination. In addition, the HRCT images of patients were processed with the optimal Atlas segmentation algorithm, the CT image characteristics of patients with mycoplasma pneumonia were analyzed, and excellent examination results were obtained. The results showed that 72 patients with mycoplasma pneumonia were examined by HRCT. A total of 37 cases were found to have unilateral lesions and 35 cases were found to have bilateral lesions. There were 19 cases in the left upper lobe, 24 cases in the left lower lobe, 21 cases in the right upper lobe, 13 cases in the right middle lobe, and 25 cases in the right lower lobe. Before treatment, the CT images of the patient showed large patchy and patchy shadows. The lung texture of the child was increased, and the shadows were striped or ground glass. The reason may be that the mycoplasma pneumonia started from bronchiole and extended to interstitial development, which led to consolidation after reaching alveoli. Due to the immature development of alveoli in children, the lung capacity is small, and the traction of alveoli on the surrounding airways was insufficient, leading to increased lung texture.^12^,^13^ This is consistent with the findings of Nakanishi et al.^14^ After the treatment, the CT images of the patients showed decreased lesion density, lesion range, and patchy shadow absorption, which suggested that antibiotics, arena corticosteroids, and other comprehensive treatments effectively killed Mycoplasma pneumoniae^15^, which was similar to the findings in a study by Matthys et al.^16^ HRCT examination of one child with mycoplasma pneumonia showed that the patient mainly had multiple patchy lesions in the anterior and posterior segments of the upper lobe tip of the left lung. It mainly presented small patchy high-density shadows with blurred edges, tree buds were seen, bronchial wall thickened and lung texture thickened and thickened, showing cable-like shadows. This was similar to the findings of Zhang et al.^17^, suggesting that the lesion had invaded the bronchioles or had parenchymal diffusion, presenting a fuzzy tree-fog shape.^18^-^20^ The results showed that HRCT based on ATLAS segmentation algorithm was effective in the diagnosis of mycoplasma pneumonia in children.

## CONCLUSION

CT diagnosis shows the pathological changes of mycoplasma pneumonia in children, and its unique imaging signs combined with clinical symptoms can improve the diagnostic accuracy of patients; Also, CT scans, especially the use of HRCT (thin-slice CT), are used for three-dimensional reconstruction. According to the changes in the characteristics of the lesion, it has great significance to evaluate the curative effect, and can be used for the clinical diagnosis of mycoplasma pneumonia and the evaluation of the curative effect.

### Author`s Contribution:

**XF** conceived the study, literature review, data analysis ,drafting the manuscript.

**NY** helped in design, drafting & critical revision of manuscript.

**JJ** takes the responsibility and is accountable for all aspects of the work in ensuring that questions related to the accuracy or integrity of any part of the work are appropriately investigated and resolved.
